# Surgical and functional outcomes of primary un-cemented total hip arthroplasty

**DOI:** 10.6026/973206300220726

**Published:** 2026-02-28

**Authors:** Manish R Shah, Shubham Nagar, Vatsal Alpesh Gupta, Chintankumar Bharatbhai Patel, Nilkumar Rakeshbhai Patel, Devanshu Garg, Ravi Undhad, Taksh V Hadvani

**Affiliations:** 1 Department of Orthopaedics, Dhiraj Hospital, Smt. B. K. Shah Medical Institute and Research Centre, Sumandeep Vidyapeeth Deemed to be University, Waghodia, Vadodara, Gujarat, India

**Keywords:** Total Hip Arthroplasty (THA), Total Hip Replacement (THR), Uncemented THR, Cementless THR, Harris Hip Score

## Abstract

There is a lacuna in literature regarding correlation between surgical and functional outcomes of un-cemented Total Hip Arthroplasty (THA). So, a prospective observational study on 30 patients was carried out for 18 months having osteonecrosis of head of femur leading to arthritis of the hip joint. We studied short term results of THA using un-cemented implants. We measured the surgical and functional outcomes by using subjective and objective parameters. We found excellent short term outcomes. Thus, though component positioning is important, our study shows that it does not affect the functional outcome in THR.

## Background:

Osteonecrosis of the femoral head (ONFH) is a chronic and multifactorial disease that begins with subtle hip discomfort and advances to total joint destruction. It often results in lifelong disability if not managed appropriately. In India, ONFH predominantly affects males in their third and fourth decades of life, with a male-to-female ratio of about 5:1. Bilateral hip involvement is seen in more than half of the cases. The condition arises due to multiple causes, the most common being chronic corticosteroid use, which accounts for approximately 37% of cases. Chronic alcohol consumption contributes to around 20% of cases, while trauma is responsible for roughly 15%. Notably, about 21% of cases are idiopathic, with no clear underlying cause. Total Hip Replacement (THR) is considered the gold standard for managing late-stage ONFH when it involves the hip joint. Several types of fixation techniques are used in THR, each with specific indications and advantages. Cemented THR has been the traditional choice, offering secure fixation, especially in elderly patients with poor bone quality. In contrast, uncemented THR, which promotes bone ingrowth for implant stability, is typically preferred in younger patients with healthy bone stock. Hybrid THR, which combines a cemented femoral stem with an un-cemented acetabular component, has shown promising results. Reverse hybrid THR, which uses a cemented acetabular cup and an uncemented femoral component, has also demonstrated encouraging results. Highly cross-linked polyethylene (HXLPE) can significantly extend the longevity of Total Hip Arthroplasty (THA). It shows no major polyethylene wear or aseptic loosening even after a 15-year follow-up. This makes HXLPE an ideal material for younger, more active patients who require durable implants [1, 2]. Remelted HXLPE offers lower wear rates compared to earlier versions and second-generation HXLPE materials, including antioxidant-infused variants, show promising mid-term outcomes. However, careful monitoring of rim fractures and other complications remains necessary and ongoing post-market surveillance will be crucial to ensuring the safety and effectiveness of these materials in the long term [3, 4]. Despite advancements in Highly Cross-Linked Polyethylene (HXLPE), wear of the polyethylene liner remains a concern. Prock-Gibbs *et al.* (2021) [5] demonstrated that HXLPE significantly reduced revision rates compared to conventional polyethylene, particularly in mid-term follow-ups. Recent studies show better 10 year survival for CoC bearing in comparison to previous reports in young individuals [6]. Studies, such as those by Knecht *et al.* (2023) [7] has shown higher revision rates due to metal debris and toxicity, with high failure rates. Wu *et al.* (2024) [8] confirmed that CoP is a reliable option, especially in less demanding populations. Boyle *et al.* (2022) [9] noted that cemented and hybrid implants provide equal survival and functional outcomes. In our prospective observational study of 30 patients, we studied the results of un-cemented THR. We studied the surgical and functional outcomes. Similarly, Kar *et al.* [21] evaluated functional outcomes using the Harris Hip Score in 25 patients undergoing primary uncemented THA, demonstrating excellent short-term results with minimal complications. Therefore, it is of interest to report the correlation between the surgical and functional outcomes.

## Materials and Methods:

A prospective observational study on 30 patients was carried out from December 2023 to June 2025. An approval from the ethical committee of our institute (approval no: SVIEC/On/Medi/BNPG 22/Dec/23/138) was taken. The patients with age more than 18 years, diagnosed having secondary arthritis of the hip treated by primary THA by un-cemented implants on one side were included. The patients with a minimum follow up of 5 months were included. A written informed consent was obtained in all the cases. The patients with age less than 18 years, those having fracture around the hip joint, revision surgery cases, bilateral operated cases, those with any other surgeries on both lower limbs were excluded. The patients with follow up less than 5 months and those who refused to participate in the study were excluded. Pre operatively all the patients were investigated by true size X-rays (AP and cross table lateral views), MRI (Magnetic Resonance Imaging) and blood parameters. The Harris Hip Score (HHS) was recorded. The implant size was decided by templates. The patients were operated by two senior joint replacement surgeons in the department with experience of more than 10 years. The patients were admitted as per the week day in different units. All patients were operated on by either postero-lateral (Southern Moore) or lateral (Hardinge) approach. The approach was decided by odd and even method by both surgeons to avoid bias. The surgeries were done as per the standard joint replacement protocol. Postoperative X-rays were recorded and computed tomography (CT) measurements were done on the same day or in couple of days before the discharge of the patient. We measured acetabular inclination, acetabular version, femoral stem version, horizontal offset and vertical offset in postoperative CT scan on both sides. We compared operated side with non-operated side. The patients were advised to touch partial weight bearing on the same day evening or next day morning. Intravenous analgesics were given for 24-48 hours followed by oral analgesics. Intravenous antibiotics (Piperacillin + Tazobactam 4.5 gm 12 hourly and Amikacin 500 mg 12 hourly) were given for 5 days followed by oral antibiotics (Cefuroxime and Clavulinic acid) for 10 days. The patients were discharged between 5-15 days (being a tertiary care hospital, we get patients from different states and it is not feasible for them to come back for the suture removal). Routine physiotherapy was advised in all the cases. The patients were discharged once the wound is good on the first dressing (usually on the 3rd day). Suture removal was done at around two weeks. The patients were called for the follow up at 1 month, 3 months, 6 months and yearly follow up. Clinical and radiological findings were recorded at each visit. The radiological evaluation of all parameters was done at each follow-up, along with evidence of polyethylene wear, osteolysis (septic/ aseptic loosening), implant migration, limb length discrepancy (LLD) and femoral subsidence and acetabular inclination. Surgical and functional outcomes were assessed during the follow-up visits using the Modified Harris Hip Score (HHS). Functional outcome was measured by the Visual Analogue Score.

## Statistical analysis:

All data were entered in Microsoft Excel and analysed using Jamovi software version 2.3.28. Descriptive statistics were presented as mean±standard deviation for continuous variables and frequencies with percentages for categorical variables. The normality of data was assessed using the Shapiro-Wilk test. For comparison of continuous variables between two groups, the independent samples t-test was used for normally distributed data. The Mann-Whitney U test was used for non-normally distributed data. A paired t-test was employed to compare pre-operative and post-operative measurements. The chi-square test was used to analyse categorical variables. The Pearson correlation coefficient was calculated to assess the relationship between continuous variables. Multiple linear regression analysis was performed to identify predictors of functional outcomes. A p-value <0.05 was considered statistically significant.

## Results:

We studied 30 patients who underwent unilateral primary total hip arthroplasty (THA) for avascular necrosis (AVN) of the femoral head with secondary arthritis of the hip joint. The mean age of the study population was 42.8±13.2 years (range: 21-70 years). A maximum number of patients (n=11, 36.7%) were in the 31-40-years age group, followed by 41-50 years (n=7, 23.3%), 51-60 years (n=7, 23.3%), 21-30 years (n=4, 13.3%) and 61-70 years (n=1, 3.3%). So, the majority were economically productive individuals, with 73.3% of patients being below 50 years of age. Correlation analysis showed no statistically significant relationship between patient age and the final Harris Hip Score (r = -0.284, p = 0.128). Out of 30 patients, 20 (66.7%) were males and 10 (33.3%) were females, showing a male predominance with a male-to-female ratio of 2:1. An independent samples t-test revealed no significant difference in final Harris Hip Scores between male (Mean = 82.5±6.1) and female (Mean = 80.5±6.6) patients (p = 0.435). In our study, 15 patients (50%) were farmers, 14 (46.7%) were laborers and 1 (3.3%) had other occupation. Idiopathic AVN was observed in 13 patients (43.3%), steroid- induced AVN in 7 patients (23.3%) and sickle cell disease in 6 patients (20%). Chronic alcoholism was in 3 patients (10%) and old healed tuberculosis in 1 patient (3.3%). We found 19 patients (63.3%) with left-sided involvement and 11 patients (36.7%) with right-sided involvement. We found bilateral involvement in 53.3% (16/30) of patients with grade I or 2A affection on one side as per the Ficat and Arlet classification. However, the patients were not having any problem on the less involved side. We included the patients who were operated on the more affected hip (unilateral). 19 patients (63.4%) on our study were operated on by postero-lateral (Southern and Moore) approach and 11 (36.6%) were operated on by lateral (Hardinge) approach. The mean duration of hospitalization was 10.7±5.1 days (range: 4-22 days). The majority of patients (n=18, 60%) had a hospital stay of 7-14 days. Only 3 patients (10%) required hospitalization for more than 14 days as shown in Table 1. Being the tertiary care hospital the most of the patients are coming from far places and they were hospitalized till suture removal. The Harris Hip Score (HHS) was used to evaluate functional outcomes at different time intervals. The mean preoperative HHS was 47.2±12.3 (range: 29-82). The immediate postoperative HHS showed improvement to a mean of 32.7±8.9 (range: 17-62). At final follow-up, the mean HHS was 81.8±6.2 (range: 70-91), demonstrating significant functional improvement as shown in Table 2. Based on the final HHS, outcomes were graded as excellent (90-100), good (80-89), fair (70-79) and poor (<70). The results showed excellent outcomes in 2 patients (6.7%), good outcomes in 17 patients (56.7%) and fair outcomes in 11 patients (36.7%). No patient had a poor outcome as shown in Table 3. Detailed ranges of movements (ROM) measurements were recorded preoperatively and postoperatively. The mean preoperative flexion was 90.3°C±13.4°C (range: 70°C-120°C), which improved to 108.7°C±8.2°C (range: 90°C-120°C) postoperatively (p<0.001). Extension improved from 8.7°C±4.1°C to 12.3°C±4.3°C (p<0.001). Abduction improved from 16.0°C±6.0°C to 22.3°C±5.1°C (p<0.001) as shown in Table 4.

CT scan measurements were performed to assess the accuracy of acetabular component positioning and were compared with opposite side as shown in Table 5. The mean acetabular inclination angle on the operated side was 47.2°C±7.9°C (range: 32.5°C-63°C), while the mean acetabular anteversion was 17.6°C±9.8°C (range: 8.9°C-51.1°C). The mean femoral version on the operated side was 10.3°C±4.7°C (range: 3°C-19.3°C), compared to 11.3°C±4.1°C (range: 3°C-18°C) on the non-operated side (p=0.367). The combined anteversion (acetabular anteversion + femoral version) was calculated for each patient. The mean combined anteversion was 27.9°C±11.2°C on the operated side, which falls within the recommended safe zone of 25°C-50°C. Horizontal and vertical offset analysis measurements are shown in Table 6. The mean follow-up duration was 27.9±5.8 weeks (range: 20-41 weeks). All patients completed a minimum follow-up of 20 weeks, with 13 patients (43.3%) having follow-up beyond 24 weeks as shown in Table 7. No major complications were observed in any of the 30 patients during the study period. There were no instances of dislocation, infection, loosening, or periprosthetic fracture. We did analysis of radiological outcomes versus HHS at the final follow-ups as shown in Table 8. We analyzed the selected patients with radiological parameters deviating from the recommended safe zones and it's relation with the final results as shown in Table 9, Table 10, Table 11- Table 12.

## Interpretation:

[1] Lower Values (Patients 4, 8): Despite being at the lower end of the normal range, they achieved good functional outcomes, suggesting adequate cup positioning.

[2] Higher Values (Patients 9, 10): Patient 9 achieved a good outcome despite higher inclination (54°C), while Patient 10, with the highest inclination (63°C), had the lowest HHS (74), suggesting potential for increased wear and instability with excessive inclination.

[3] The negative correlation suggests that extremely high acetabular inclination may compromise functional outcomes.

[4] Within Optimal Range (Patients 2, 4): Both achieved good outcomes with combined versions of 47.8°C and -0.6°C, respectively.

[5] Above Optimal Range (Patients 3, 10): Patient 3 achieved an excellent outcome (91 HHS) despite a high combined version, while Patient 10 had a fair outcome, suggesting individual variation in tolerance.

[6] Below Optimal Range (Patients 6, 9): Both achieved good outcomes despite lower combined versions, indicating compensatory mechanisms.

## Statistical analysis summary:

## Correlation analysis:

## Pearson correlation analysis revealed:

[1] Negative correlation between age and final HHS (r=-0.284, p=0.128)

[2] Positive correlation between preoperative HHS and final HHS (r=0.412, p=0.024)

[3] No correlation between hospital stay and final outcomes (r=-0.087, p=0.648)

## Regression analysis:

Multiple linear regression analysis identified preoperative HHS as the only significant predictor of final functional outcome (β=0.412, p=0.024), while age, gender, etiology and surgical approach were not significant predictors.

## Discussion:

The mean age of 42.8 years in our study is consistent with the global trend of AVN affecting younger individuals compared to osteoarthritis. This finding aligns with study by Wade and Shah [10], which reported a mean age less than 40 years for AVN patients. Similarly, Kar *et al.* [21] reported a mean age of 53.56 years in their study of 25 patients undergoing uncemented THA, indicating that this procedure is also suitable for an older demographic with good bone quality. The younger age profile has significant implications for implant selection and long-term survivorship considerations. The male predominance (66.7%) observed in our study corroborates the findings of Li *et al.* [11], who reported90.9% males in their study. This gender distribution may be attributed to the higher prevalence of risk factors such as alcohol consumption and occupational hazards in males. Comparatively, studies from developed nations report different occupational distributions. Reports by Birajdar *et al.* [12], who documented increased incidence of coagulopathy and need for higher doses of anticoagulants following the coronavirus disease of 2019(COVID-19) THR, attributed to high-dose corticosteroid use. Our findings support the need for judicious steroid use and close monitoring of patients receiving corticosteroids. Sickle cell disease accounted for 20% of cases in our study. Mohabey *et al.* [13] reported sickle cell AVN as considerable challenging condition for the patients for THR. Sirignani *et al.* [14] reported 40% decline instability rate following primary THR. The absence of dislocations in our study, regardless of approach, may be attributed to appropriate patient selection, surgical technique and postoperative rehabilitation protocols. All patients in our study received uncemented implants, reflecting the current preference for biological fixation in younger patients with good bone quality. This approach is supported by many studies [11, 15, 16], which demonstrate superior long-term survivorship of uncemented implants in patients under 55 years. Our results demonstrate that with appropriate patient selection and surgical technique, uncemented fixation can achieve excellent outcomes even in the setting of AVN. The mean Harris Hip Score improvement from 47.2 to 81.8 represents a clinically significant enhancement in function. This 34.6-point improvement exceeds the minimal clinically important difference (MCID) of 18 points. These findings are comparable to Kar *et al.* [21], who reported HHS improvement from a preoperative median of 50 to a postoperative median of 91 at 12 months in their 25-patient cohort, with 72% achieving excellent outcomes. Our results are lower to those reported by Sidhu *et al.* [17], who found a mean HHS of 90.4+7.3 in their series of uncemented THA. This difference may be attributed to our shorter follow-up period. However, the absence of poor outcomes in our series is encouraging and suggests appropriate patient selection and surgical execution. Our study revealed better outcomes in younger patients, though not statistically significant. The lack of statistical significance in our study may be due to the relatively small sample size and homogeneous age distribution. The mean acetabular inclination of 47.2°C falls within Lewinnek's safe zone of 40°C ± 10°C, though slightly on the higher side. This is consistent with contemporary understanding that the "safe zone" concept may be overly simplistic. The cup placement in relation to transverse acetabular ligament (TAL) is reproducible and reliable method for THR [18].

The combined anteversion of 27.9°C falls within the recommended range of 25°C-50°C. This concept recognizes the interdependence of acetabular and femoral component positioning. We did all surgeries by either Hardinge (lateral) or Southern-Moore (postero-lateral) approach. Recent studies comparing direct anterior approach (DAA) and posterior approaches show that the results are comparable [19]. Ansari *et al.* compared direct lateral and posterior approaches [20]. They reported abductor and extensor mechanism weakness upto 6 weeks after THR in their study. We have not found such problem in our series. Our results support the importance of assessing combined anteversion rather than isolated component measurements. The absence of major complications in our series is remarkable but must be interpreted cautiously, given the relatively short follow-up period. This finding is consistent with Kar *et al.* [21], who reported minimal complications in their series (two superficial infections, one sciatic nerve injury, and one dislocation in 25 patients), demonstrating that uncemented THA can be performed safely with appropriate surgical technique. Selection of a wide range of patients, detailed analysis of clinical and radiological parameters and prospective nature can be considered strengths of our study. A small sample size, shorter follow-up period, single centre study and absence of control group can be considered weakness of our study. The studies with long term follow-up, randomized control trials, multi-centre studies and those involving a large number of patients are required to have a better conclusion.

## Conclusion:

We show that un-cemented total hip arthroplasty provides excellent short-term functional outcomes for patients with AVN of the femoral head. The significant improvements in HHS, range of motion and the supporting radiological parameters, for each patient, combined with the absence of major complications; support THA as an effective treatment modality. Though component positioning is important, our study shows that it does not affect the functional outcome in THR.

## Advancement to Knowledge:

The article provides correlation between surgical and functional outcomes of primary un-cemented THR by using standard parameters. It shows that accuracy of implant placement definitely maters but functional results may not be always affected by it.

## Figures and Tables

**Table 1:** Showing hospital stay

**Duration (Days)**	**Number of Patients**	**Percentage (%)**
≤7	9	30
14-Aug	18	60
>14	3	10
Total	30	100

**Table 2:** Showing harris hip score at different intervals

**Time Interval**	**Mean HHS**	**SD**	**Range**	p-value*
Preoperative	47.2	12.3	29-82	-
Postoperative	32.7	8.9	17-62	<0.001
Final Follow-up	81.8	6.2	70-91	<0.001
*Paired t-test comparing with preoperative values				

**Table 3:** Harris Hip Score grade distribution at final follow-up

**Grade**	**Score Range**	**Number of Patients**	**Percentage (%)**
Excellent	90-100	2	6.7
Good	80-89	17	56.7
Fair	70-79	11	36.7
Poor	<70	0	0
Total	-	30	100

**Table 4:** Showing range of movement comparison

**Movement**	**Pre-operative (Mean±SD)**	**Post-operative (Mean±SD)**	p-value*
Flexion	90.3°C ± 13.4°C	108.7°C ± 8.2°C	<0.001
Extension	8.7°C ± 4.1°C	12.3°C ± 4.3°C	<0.001
Abduction	16.0°C ± 6.0°C	22.3°C ± 5.1°C	<0.001
Adduction	12.3°C ± 6.8°C	7.3°C ± 6.2°C	<0.001
Internal Rotation	5.0°C ± 5.8°C	7.0°C ± 5.3°C	0.023
External Rotation	17.0°C ± 6.2°C	24.0°C ± 5.1°C	<0.001
*Paired t-test			

**Table 5:** Showing acetabular component position after surgery

**Parameter**	**Operated Side (Mean±SD)**	**Non-operated Side (Mean±SD)**	p-value*
Inclination	47.2°C ± 7.9°C	47.7°C ± 7.3°C	0.782
Anteversion	17.6°C ± 9.8°C	15.6°C ± 5.8°C	0.324
*Paired t-test

**Table 6:** Showing comparison of horizontal and vertical offsets

**Parameter**	**Operated Side**	**Non-operated Side**	**Difference**	p-value*
**Horizontal Offset (cm)**				
Mean ± SD	5.82 ± 0.69	5.98 ± 0.86	-0.16 ± 0.58	0.147
Range	4.9-7.1	4.7-7.5		
**Vertical Offset (cm)**				
Mean ± SD	4.15 ± 0.66	4.12 ± 0.53	0.03 ± 0.44	0.724
Range	2.9-5.5	3.3-4.9		
*Paired t-test

**Table 7:** Showing follow up duration

**Duration (Weeks)**	**Number of Patients**	**Percentage (%)**	**Mean HHS ± SD**
24-29	20	66.70%	81.4 ± 6.8
30-34	7	23.30%	82.0 ± 5.4
≥35	3	10.00%	83.7 ± 4.2
Total	30	100%	81.8 ± 6.2

**Table 8:** Showing correlation between radiological parameters and final HHS

**Radiological Parameter**	**Pearson's r**	**p-value**
Acetabular Inclination (Operated)	-0.142	0.455
Acetabular Anteversion (Operated)	-0.087	0.648
Femoral Version (Operated)	0.156	0.411
Combined Anteversion (Operated)	0.038	0.842
Horizontal Offset (Operated)	0.213	0.259
Vertical Offset (Operated)	-0.098	0.607

**Table 9:** Showing relationship between operated acetabular inclination and final HHS in selected patients (Statistical Analysis: Pearson correlation r = -0.607, p = 0.280)

**Patient No.**	**Name**	**Acetabular Inclination (°C)**	**Final HHS**	**Interpretation**
2	JK	37.7	85	Within normal range (35-50°C), Good outcome
4	BH	32.5	81	Below normal range, Good outcome
8	KH	38	82	Within normal range, Good outcome
9	SW	54	84	Above normal range, Good outcome
10	NK	63	74	Significantly elevated, Fair outcome

**Table 10:** Showing combined anteversion (acetabular + femoral version) and final Harris Hip Score [Statistical Analysis: Pearson correlation r = -0.182, p = 0.727 Optimal Range: 25-50°C (McKibbin's safe zone)]

**Patient No.**	**Name**	**Acetabular Version (°C)**	**Femoral Version (°C)**	**Combined Version (°C)**	**Final HHS**	**Interpretation**
2	JK	30	17.8	47.8	85	Optimal range, Good outcome
3	MP	51.1	11.6	62.7	91	Above optimal, Excellent outcome
4	BH	-15.6	15	-0.6	81	Optimal range, Good outcome
6	AS	8.9	9.7	18.6	83	Below optimal, Good outcome
9	SW	13.1	3.6	16.7	84	Below optimal, Good outcome
10	NK	35.1	19.3	54.4	74	Above optimal, Fair outcome

**Table 11:** Showing horizontal offset restoration and functional outcomes

**Patient No.**	**Name**	**Operated HO (cm)**	**Non-operated HO (cm)**	Difference* (cm)	**Final HHS**
1	SR	4.9	7.2	2.3	84
2	JK	5.7	6	0.3	85
3	MP	6.5	5.3	-1.2	91
4	BH	6.6	6.7	0.1	81
5	DR	7	6.5	-0.5	87
6	AS	6.6	7.2	0.6	83
7	BR	5.8	5	-0.8	80
8	KH	7	5.4	-1.6	82
9	SW	6.2	6.3	0.1	84
10	NK	6.2	7.4	1.2	74
[*Positive values indicate
under-restoration and Negative
values indicate over-restoration.
Statistical Analysis: Pearson
correlation r = -0.335,
p = 0.346. Mean Difference:
+0.01±1.3 cm (Range: -1.6 to +2.3 cm)]

**Table 12:** Showing vertical offset restoration and functional outcomes

**Patient No.**	**Name**	**Operated VO (cm)**	**Non-operated VO (cm)**	Difference* (cm)	**Final HHS**
1	SR	3.5	4.6	1.1	84
2	JK	3.8	4.5	0.7	85
3	MP	4.3	4.9	0.6	91
4	BH	4.9	4.1	-0.8	81
5	DR	3.6	3.5	-0.1	87
6	AS	5.3	3.8	-1.5	83
7	BR	4.2	4.6	0.4	80
8	KH	2.9	3.4	0.5	82
9	SW	4	3.8	-0.2	84
10	NK	5	4.8	-0.2	74
[*Positive values indicate
under-restoration,
Negative values indicate
over-restoration
Statistical Analysis:
Pearson correlation
r = +0.267, p = 0.456.
Mean Difference: +0.05±0.8 cm
(Range: -1.5 to +1.1 cm)]
